# Evaluation of Resting Energy Expenditure in Subjects with Severe Obesity and Its Evolution After Bariatric Surgery

**DOI:** 10.1007/s11695-021-05578-5

**Published:** 2021-08-03

**Authors:** Enzamaria Fidilio, Marta Comas, Miguel Giribés, Guillermo Cárdenas, Ramón Vilallonga, Fiorella Palma, Rosa Burgos Peláez, Rafael Simó, Andreea Ciudin

**Affiliations:** 1 Vall d’Hebron Institut de Recerca, Universitat Autònoma de Barcelona (VHIR-UAB), Barcelona, Spain; 2 Endocrinology and Nutrition Department, Hospital Universitari Vall Hebron, Pg Vall d´Hebron 119-129, 08035 Barcelona, Spain; 3 Universitat de Vic (UVic)- Human Nutrition and Dietetics, Vic, Spain; 4 Bariatric and Metabolic Surgery Department, Hospital Universitari Vall Hebron, Barcelona, Spain; 5 CIBER de Diabetes y Enfermedades Metabólicas Asociadas, Instituto de Salud Carlos III, Barcelona, Spain; 6 Diabetes Research and Metabolism Unit, Institut de Recerca Hospital Universitari Vall d’Hebron (VHIR), Pg. Vall d’Hebron 119-129, 08035 Barcelona, Spain

**Keywords:** Bariatric surgery, Severe obesity, Resting energy expenditure

## Abstract

**Purpose:**

One major determinant of weight loss is resting energy expenditure (REE). However, data regarding REE is scarce in patients with severe obesity (SO)—BMI>50kg/m^2^. Most studies used equation in order to estimate REE and not indirect calorimetry (IC) (gold standard). Additionally, there is no reliable data on the impact of bariatric surgery (BS) on REE.

**Objectives:**

(a) To evaluate the REE in patients with SO; (b) to compare REE measured by IC (mREE) to that calculated by Mifflin St-Jeor equation (eREE); (c) to evaluate the impact of BS on REE and the relationship with evolution post-BS.

**Material and Methods:**

Single-center observational study including consecutive patients with SO between January 2010 and December 2015, candidates for BS. mREE was determined at baseline, and 1 and 12 months post-BS by IC, using a Vmax metabolic monitor.

**Results:**

Thirty-nine patients were included: mean age 46.5±11.77 years, 64.1%women. Preoperative mREE was 2320.38±750.81 kcal/day. One month post-BS, the mREE significantly decreased (1537.6 ± 117.46 kcal/day, *p* = 0.023) and remained unchanged at 12 months (1526.00 ± 123.35 kcal/day; *p* =0.682). Reduction in mREE after the BS was a predictor of reaching successful weight loss (nadir) and weight regain (5 years follow-up) (AUCROC of 0.841 (95%CI [0.655–0.909], *p*=0.032) and AUCROC of 0.855 (95% CI [0.639–0.901]), *p*= 0.027, respectively). eREE was not valid to identify these changes.

**Conclusion:**

In patients with SO, a significant reduction of mREE occurs 1 month post-BS, unchanged at 12 months, representing the major conditioning of successful weight loss and maintenance post-BS.

**Graphical abstract:**

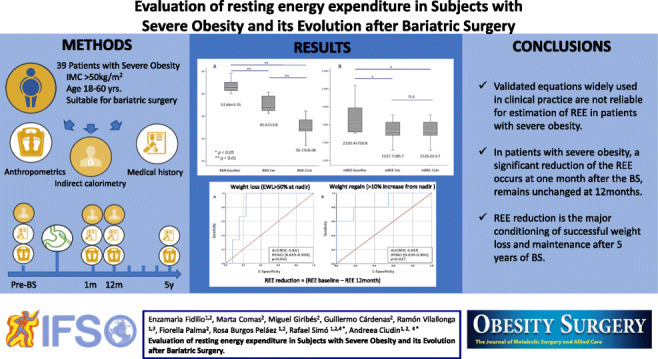

## Introduction

The physiology of weight gain and weight loss is complex, multifactorial, and by far to be completely elucidated. A recent systematic review identified 124 determinants of weight loss maintenance. Of those, reducing energy intake, increasing energy expenditure, and monitoring behaviors showed the strongest level of evidence [1]. One of the major determinants is the balance between energy intake and energy expenditure. Weight variations are associated with variations in total energy expenditure (TEE) [2]. TEE is influenced by factors such as age, gender, weight, body composition, diet, and physical activity [3]. TEE is defined as the amount of heat energy used by the human body for daily physiological functions and is divided into 3 main components: (a) resting energy expenditure (REE)—accounting for around 70% of TEE; (b) diet-induced thermogenesis (DIT); and (c) activity energy expenditure (AEE) [4].

Historically, several methods have been developed for assessing TEE. However, each approach has its advantages and disadvantages. If the purpose is to assess free-living TEE, doubly labelled water (DLW) is recommended. DLW provides information on TEE for a 4–20-day period, likely to reflect the normal energy requirement of individuals. DLW is proven to be safe and useful in all age groups and in several clinical settings. On the other hand, it is highly expensive, and proper equipment and specialized expertise are required to analyze isotope concentration in body fluids by mass spectrometry [5].

Direct calorimetry measures total heat loss from the body while the participant is isolated in a thermally controlled chamber. Although very accurate, it is unpractical for measuring TEE in a free-living population context. On the other hand, indirect calorimetry measures CO_2_ production and VO_2_ consumed in a controlled environment (closed-circuit) to calculate the amount of energy expended. It should be noted that if performed in a resting state, IC will allow the measurement of REE, which is not provided by other techniques. For this reason, IC is considered the gold standard for REE measure [4]. Additionally, the technique for REE measure is time-saving and requires minimal training, making it feasible and practical for study populations. Furthermore, in order to assess exercise metabolism, open-circuit portable indirect calorimetry techniques are more suitable. More recently, heart rate monitoring portable devices may be useful for assessment of physical activity rather than TEE. Finally, questionnaires of activity recall and motion sensors, such as pedometers and accelerometers, may have a role in evaluating interventions aimed at increasing physical activity; instead, its use to quantify REE is very limited [5].

Some data in the literature suggested that REE is increased in patients with morbid obesity [6]. Reliable data on REE in these cases is necessary to personalize calorie intake in order to assure a safe and effective weight loss and, more importantly, weight maintenance after successful weight loss. Nevertheless, REE is calculated in the daily clinical practice by means of estimation equations. Although widely used in clinical settings, it should be noted that these equations were validated based on data from healthy normoweighted subjects. These estimations are not always accurate for REE in subjects with overweight or obesity [7]. Actual published evidence is reflecting great disparities between predicted and measured energy expenditure values in patients with obesity [8–10]. Additionally, at present, there is no reliable data regarding their accuracy in estimating REE in patients with severe obesity (SO).

Bariatric surgery (BS) has proven to be an effective treatment for obesity resulting in sustainable and substantial weight loss and improvement of related comorbidities [11]. Furthermore, BS was proven to be safe and effective in patients with SO at short-medium follow-up, representing the preferred treatment for obesity in these patients [12]. Some authors suggested that BS can modify the REE and have proposed that the greater long-term success of BS as a treatment for obesity could be partially explained by the effects of BS on REE [13]. Nevertheless, others have found no influence of REE on outcomes after BS, rather a compensatory adaptive thermogenesis mechanism that occurs in response to a decreased energy intake [14]. Whether the changes in REE after BS act as determinants of weight loss maintenance is still under investigation. One possible mechanism comes from evidence in rodents. In obese mice, bariatric surgery seems to increase brown adipose tissue activity postoperatively resulting in increased energy consumption and decreased respiratory exchange frequency. These effects deteriorated when mice experienced weight regain 8 weeks after surgery [15]. In humans, evidence supporting a “browning” of adipose tissue after BS is increasing. However, evidence in the literature is contradictory [16].

It should be noted that around 20–25% of patients that undergo BS do not achieve successful weight loss [17], or, more importantly, about 30–35% fail to maintain weight loss [18], experiencing significant weight regain starting from 3 years after the BS [19]. Additionally, weight loss is often less significant than would be expected for a given degree of caloric restriction or BS technique [20]. While it is clear that individuals differ in the susceptibility to weight loss (and their subsequent ability to sustain this lower body weight), robust predictors of response to a weight loss intervention remain unclear. Data regarding REE is very scarce in patients with SO, and practically there is no reliable data on the impact of BS on REE in this population [21].

On these bases, the aims of the present study were as follows: (a) to evaluate the REE in patients with SO by means of the *gold standard* method (IC); (b) to compare the values of the REE measured by IC (mREE) to the estimated value calculated by equation (eREE); (c) to evaluate the impact of BS on the REE and the relationship with the evolution post-BS (in terms of weight loss, weight regain, and resolution of comorbidities).

## Material and Methods

A single-center observational study including consecutive patients with SO and BMI >50 kg/m^2^ attended the Morbid Obesity Unit of a third-level university hospital (Vall d´Hebron University Hospital) that had performed IC between January 2010 and December 2015. The study was approved by the Ethics Committee of our site and conducted following the Strengthening the Reporting of Observational Studies in Epidemiology guidelines and the statements of the Declaration of Helsinki. The patients signed the informed consent form prior to inclusion in the study.

All the patients underwent a complete medical history, anthropometric evaluation, and IC at baseline, 1 month, and 12 months after the BS.

Inclusion criteria: (a) signed informed consent; (b) age between 18 and 60 years (the limits for BS at our site); (c) BMI >50kg/m^2^; (d) eligible for BS according to the *standard of care* protocol at our site.

Exclusion criteria: (a) eating disorders; (b) endocrine disease or treatment with potential influence on the REE (egg: systemic corticosteroids, untreated hyper/hypothyroidism); (c) severe illness that can influence the outcomes; (d) unable to perform the follow-up visits post BS at our site; (e) other surgery than sleeve gastrectomy (SG) or Roux-en-Y gastric bypass (RYGB); (f) second-step BS or revision surgery.

Procedures and variables collected for the study:

### Clinical and Anthropometric Variables

#### Collected at Baseline

Age, gender, weight (kg), height (m), BMI (kg/m^2^), excess of body weight (EBW) (kg), presence of comorbidities related to obesity. Excess body weight (EBW) was defined as follows: actual weight − ideal body weight (IBW) based on BMI 25 kg/m^2^.

#### Collected During Follow-up 1 Month, 12 Months, and 5 Years After the BS

Weight (kg), BMI (kg/m^2^), percentage of excess of weight loss (%EWL), total weight loss (TWL), percentage of total weight loss (%TWL), and evolution of related comorbidities. Weight and BMI nadir were considered the minimum values reached after the BS; %EWL, TWL, and %TWL were calculated following standardized outcome reporting guidelines [22]. The post-BS weight regain was defined as a 10% regain of the minimal weight after BS, as previously described [23].

### Energy Expenditure Determination (REE) Variables

Collected at baseline, 1 month, and 12 months after the BS:

#### Estimated Equations (eREE)

Although the Harris-Benedict Equation (HBE) [24] is widely used in clinical practice, it appears to be less accurate when compared to the Mifflin-St Jeor equation (MSJ) in patients with obesity [13]. In this study, we used the Mifflin-St Jeor Equation (MSJ) [25]: 9.99*weight (kg) + 6.25*height (cm) − 4.92 * age + 166 * sex (M = 1; F = 0) −161.

#### Indirect Calorimetry (mREE)

IC was performed in supine position, on a neutral environment, and after resting for at least 20 min, using a Vmax 29 (Sensor Medics, Yorba Linda, CA, USA) portable metabolic monitor, available at our site. After the resting period, 15–20 min of calorimetric data was collected. The first 5 min of data was excluded in all cases. The equipment was calibrated prior to each measurement. The patients were instructed to avoid stimulating drinks, cigarette smoking, and exercise 24 h prior to and to be fasting at least 8 h prior to the performance of IC. Oxygen consumption (VO_2_), carbon dioxide production (VCO_2_), respiratory quotient (RQ), and resting energy expenditure (mREE) are generated in the final report.

### Statistical Analyses

IBM SPSS statistical software version 24 was used. Continuous variables are expressed as means ± standard deviation (SD) for normal distributed variables and median ± interquartile range (IQR) for non-normal distributed variables. Categorical variables are expressed with percentages. For differences between groups in continuous variables, Student’s *t* test or U-Mann-Whitney test was used while *χ*^2^ was used for categorical variables. For differences between 3 and more time points, repeated-measures ANOVA was used; if differences were found, a post hoc pairwise comparison was performed. Differences in weight loss rates at nadir and weight regain rates 5 years after surgery with predetermined definitions were explored using descriptive statistics. Correlation analysis was used to explore the associations between demographics (i.e., age, gender, and preoperative BMI), type of surgery (SG vs RYGB), presence of comorbidities, REE variables, and weight loss at nadir and weight regain 5 years after surgery according to the different definitions. Akaike Information Criterion (AIC)–based backward selection was used to remove insignificant terms from an initial model containing all the candidate predictors*.* A *p*-value < 0.05 was considered statistically significant.

## Results

**Fig. 1:**
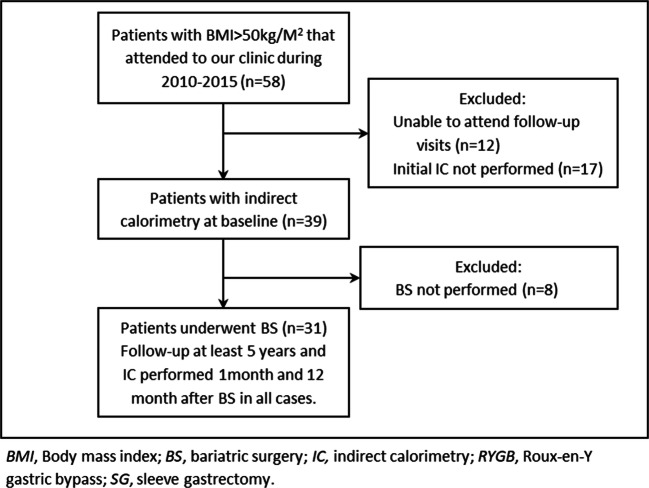
Flowchart of the inclusion of the patients in the study. BMI, body mass index; BS, bariatric surgery; IC, indirect calorimetry; RYGB, Roux-en-Y gastric bypass; SG, sleeve gastrectomy

A total of 39 patients with SO and BMI >50 kg/m^2^ were included in the study as detailed in Figure 1. The baseline clinical and demographical characteristics of the patients are shown in Table 1. Measured REE was 2320.38 ± 750.81 kcal/day and significantly different to MSJ equation estimation (1994.44 ± 463.41 kcal/day, *p*= 0.035). Additionally, mREE directly correlated with initial weight; initial BMI and EW (0.792, 0.451, and 0.795 respectively *p* < 0.0001) indirectly correlated with age (−0.769, *p* < 0.0001). We found no difference in mREE between patients with or without associated comorbidities, including when stratified for number of comorbidities. As expected, mREE was significantly different among men and women. Measured REE was higher in men compared to that in women (2761.0±122.0 kcal/day vs. 1964.0 ±622.0 kcal/day, *p* <0.001).

**Table 1:** Baseline characteristics of patients with severe obesity

***Demographics***		*n* = 39
Gender, females, % (*n*)		64.10 (25)
Age (years), mean (SD)		46.5 ± 11.7
Initial weight (kg), mean (SD)		149.3 ± 30.36
BMI (kg/m^2^), mean (SD)		56.2 ± 5.6
EW (kg), Mean (SD)		83.1 ± 22.3
***Obesity-associated comorbidities***		
Type 2 diabetes, % (*n*)		30.8 (12)
Hypertension, % (*n)*		38.5 (15)
Dyslipidemia, % (*n*)		17.9 (7)
OSA, % (*n*)	Absent Mild Moderate Severe	20.5 (8) 15.4 (6) 5.1 (2) 59 (23)
Number of obesity-related comorbidities, % (*n*)	None 1 2 3 4	10.3 (4) 17.9 (7) 35.9 (14) 20.5 (8) 15.3 (6)

*BMI* body mass index, *EW* excess of weight, *NAFLD* non-alcoholic fatty liver disease, *OSA* obstructive sleep apnea

One of the variables reported in the IC is the RQ (ratio of the amount of carbon dioxide produced to the amount of oxygen consumed), used to calculate rates of carbohydrate versus fat used to support energy metabolism. In this regard, when a molecule of glucose is metabolized, the RQ has a value of 1.0. Similarly, when one molecule of fat (tripalmitin) is completely metabolized, the RQ is 0.71 [26]. In our cohort, RQ-baseline was 0.81±0.1, suggesting a fat oxidation–prone metabolism. In this regard, we found a negative statistical significant correlation with initial weight, initial BMI, and EW (*r* =−0.390, *p*= 0.01; *r*= −0.313, *p*= 0.05 and −0.423, *p*= 0.007, respectively)

### Evolution After BS

As reflected by Figure 1, 31 patients underwent BS and at least 5 years of follow-up: 22.6% underwent RYGB and 77.4% underwent SG. As per protocol, the SG is the recommended technique in almost all of the patients with SO. The data at 5 years follow-up is shown in Table 2. Patients achieved the minimum weight after the BS (nadir) after a mean follow-up of 17.1 ± 4.8 months after the BS: weight 80.2±20.5 kg, BMI 33.2±10.5 kg/m^2^. At this point, 87.01% (27/31) of the patients achieved >20%TWL and 80.6% (25/31) met a >50%EWL, regardless of the age, gender, or type of surgery.

**Table 2:** Follow-up subgroup characteristics

***N=31***		***Baseline***	***1-year FU***	***Nadir***	***5-years FU***
Age (years)		50.44± 7.52			
Sex, females, % (*n*)		67.7 (21)			
Type of surgery, % (*n*)		SG 77.4 (24) RYGB 22.6 (7)			
Weight (kg)		135.98±20.11^a^	92.08±23.22^a^	88.35±24.12^a^	94.37±24.67^a^
BMI (kg/m^2^)		53.66±3.35^a^	36.13±6.06^a^	34.60±6.29^a^	37.04±6.02^a^
EW (kg)		72.51±11.98^a^	27.61±18.54^a^	24.89±18.54^a^	30.91±18.36^a^
***Comorbidities***					
Type 2 diabetes, % (*n*)		45.2 (14)	6.4 (2)	6.4 (2)	19.3 (6)
Hypertension, % (*n)*		38.7 (12)	12.9 (4)	12.9 (4)	12.9 (4)
Dyslipidemia, % (*n*)		25.8 (8)	6.4 (2)	6.4 (2)	12.9 (4)
OSA,% (*n*)	Absent Mild Moderate Severe	45.2 (14) 12.9 (4) 32.2 (10) 12.9 (4)	61.3 (19) 32.2 (10) 6.4 (2) 0 (0)	61.3 (19) 32.2 (10) 6.4 (2) 0 (0)	51.6 (16) 22.5 (7) 12.9 (4) 6.4 (2)
***Weight loss***					
Percent of total weight loss (%TWL) %TWL> 20, % (*n*)			32.34±12.06^a^ 83.87 (26)	35.13±12.58^a^ 87.01 (27)	30.74± 12.49^a^ 74.1 (23)
Percent excess weight loss (%EWL) %EWL >50, % (*n*)			60.44±20.76^a^ 64.5 (20)	65.67±21.78^a^ 80.6 (25)	57.32 ± 21.58^a^ 67.7 (21)

*BMI* body mass index, *EW* excess of weight, *FU* follow-up, *OSA* obstructive sleep apnea, *RYGB* Roux-en-Y gastric bypass, *SG* sleeve gastrectomy. Continuous variables expressed in mean ± SD. ^a^Repeated-measures ANOVA, *p* < 0.001

At 5-year follow-up, weight was 94.37±24.67 kg and BMI 37.04±6.02 kg/m^2^, significantly increased from nadir (*p*<0.001), representing a significant weight regain in 32.25% (10/31) of the patients.

### Changes in REE After BS

**Figure 2:**
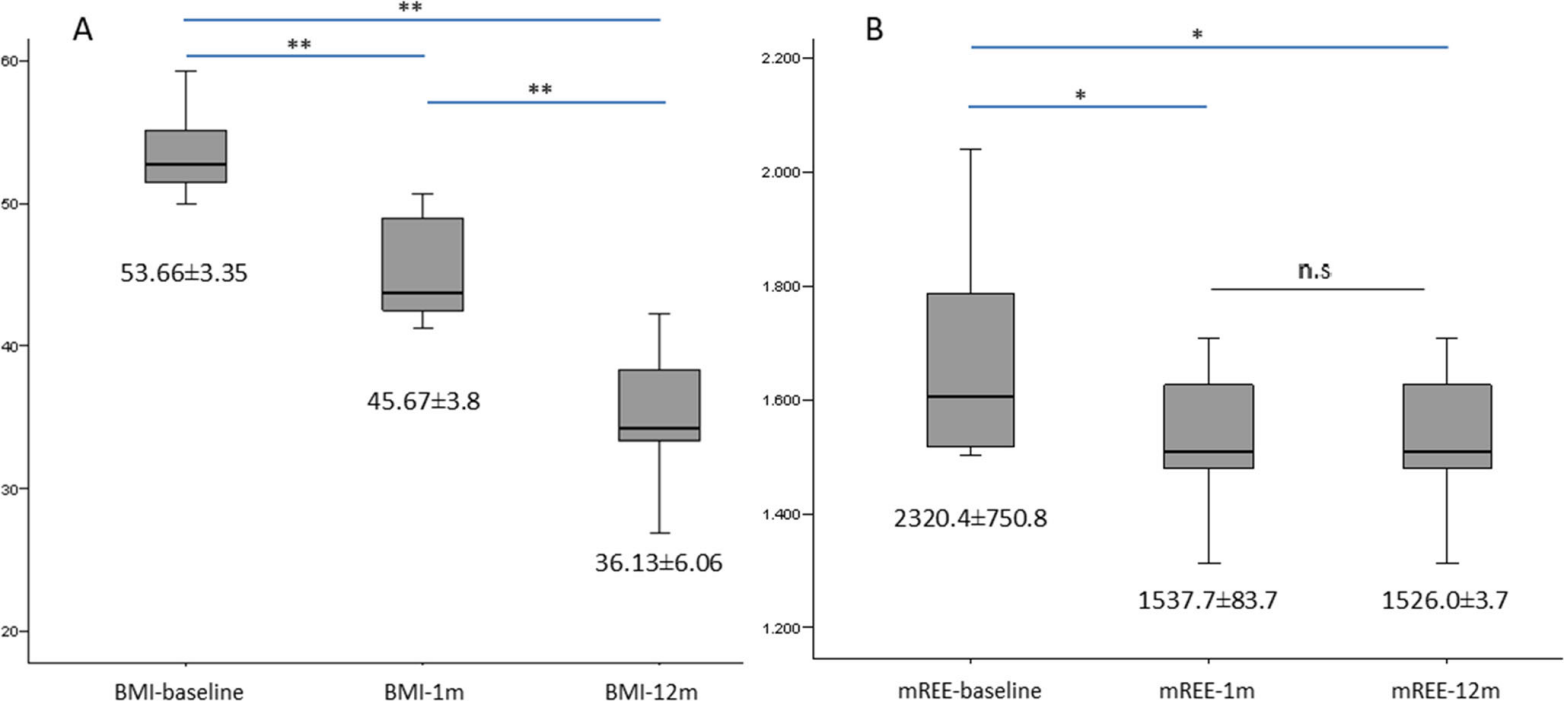
Changes in measured resting energy expenditure and body mass index before and after bariatric surgery. BMI, body mass index; mREE, measured resting energy expenditure; 1m, 1 month after bariatric surgery; 12m, 12 months after bariatric surgery. Repeated-measures ANOVA for: **A** BMI and **B** mREE before and after BS, *p* < 0.001. Results after Bonferroni correction are indicated if significant differences were found. *p < 0.05; **p < 0.01

We found a significant reduction in mREE at least 1 month after the BS, achieving levels comparable to those of the Spanish population with normal weight [35], despite presenting BMI in morbid obesity range (BMI-1m after BS 45.67±3.80kg/m^2^). The mREE-12m remained significantly unchanged after the initial significant “drop-down” 1m after BS, while BMI-12m continued to significantly reduce (36.13±6.06kg/m^2^, *p*<0.0001). Figure 2 and Table 3 show the evolution of the IC parameters after the BS. We found no statistical significant differences among techniques in REE at any time point.

**Table 3:** Changes in energy metabolism

***N=31***	***Baseline***	***1 month after BS***	***12 months after BS***	***p value*** *ǂ*
mREE (kcal/day)	2320.4 ± 750.8^a^	1537.7 ± 83.7^a, b^	1526.0 ± 3.7b	0.006
MSJ (kcal/day)	1994.4 ± 463.4^a^	1789.1 ± 307.5^a^	1551.1 ± 349.8^a^	0.001
RQ (V_CO_^2^/ V_O_^2^)	0.81 ± 0.13	0.79 ± 0.08	0.81 ± 0.07	n.s

*mREE* measured resting energy expenditure, *MSJ* Mifflin St-Jeor equation, *RQ* respiratory quotient, *V*_*O*_^*2*^ oxygen consumption (ml/min^−1^), *V*_*CO*_^*2*^ carbon dioxide production (ml/min^−1^). Continuous variables expressed in mean ± SD

ǂRepeated-measures ANOVA

^a^Bonferroni correction *p* <0.005

^b^Bonferroni correction *p* =n.s

An inverse correlation was found between initial EW and mREE-1m and mREE-12m (*r* = −0.714, *p* = 0.047 and *r* = −0.681, *p* = 0.014, respectively). However, mREE-1m and mREE-12m did not correlate with any other weight-related variables (i.e., initial weight, 1m-weight, 1m-EW, nadir weight, nadir EW). An indirect correlation was observed between mREE-1m and mREE-12m and RQ-1m and RQ-12m, respectively, but not with mREE and RQ at baseline.

Although we found a significantly difference between gender at mREE-baseline, these differences were no longer significant after BS, while MSJ showed differences between gender in all three time points, as reflected by Table 4.

**Table 4:** Differences among gender in mREE and MSJ across three time points

*N=31*		*REE-baseline*	*REE-1m*	*REE-12m*
mREE (kcal/day)	Female	1769.36±245.81^a^	1529.45±100.74	1526.0±95.55^b^
	Male	2561.0±449.40^a^	1555.73±40.40^b^	1548±72.32
MSJ (kcal/day)	Female	1778.36±97.92^a^	1604.73±97.62^a^	1393.91±107.31^a, b^
	Male	2469.80±245.79^a^	2194.80±177.32^a, b^	1896.80±461.10a
RQ	Female	0.92±0.19^a^	0.81±0.02	0.81±0.09
	Male	0.78±0.01^a^	0.79±0.10	0.80±0.01

^a^Significant difference between women and men *p* <0.05 ^b^Significant difference between gender and mREE or MSJ *p*<0.05

**Figure 3:**
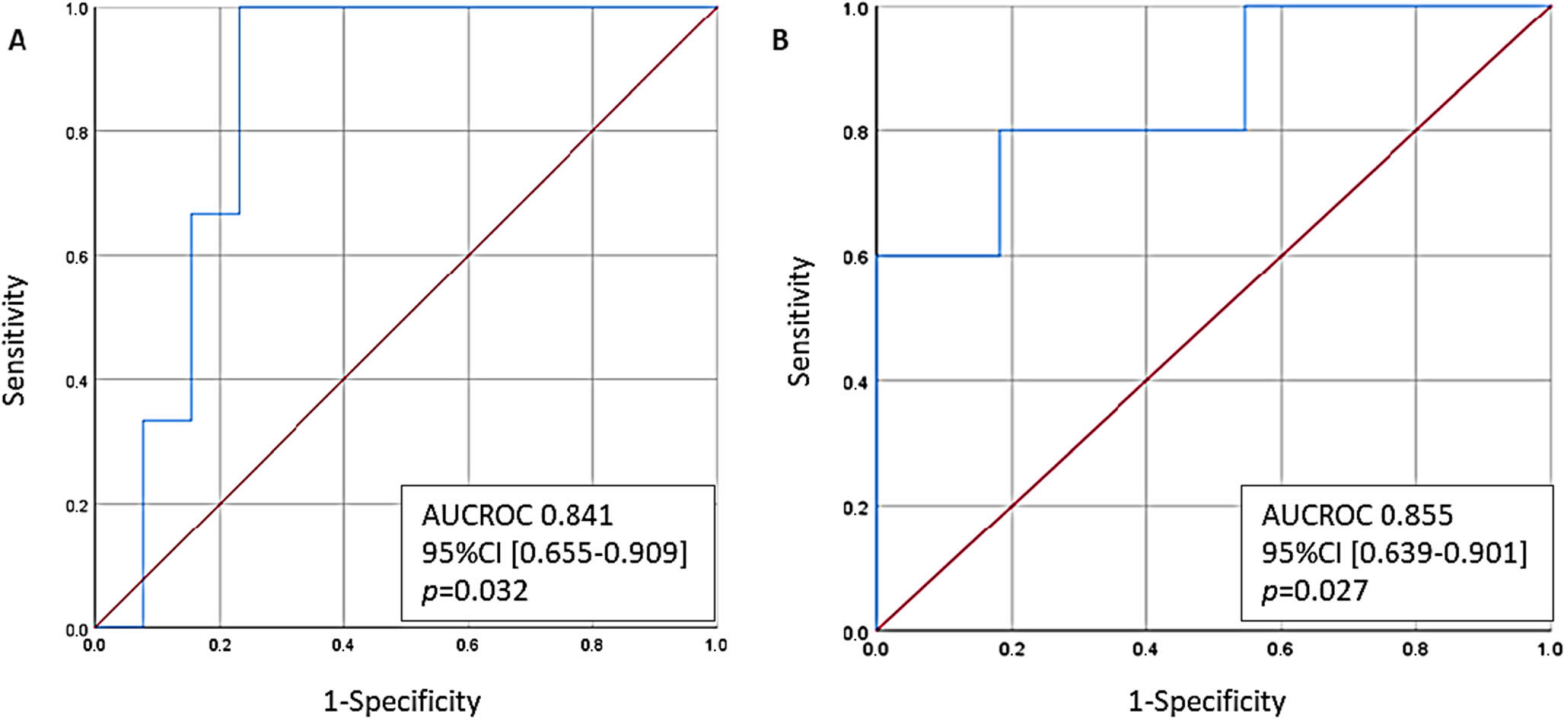
The predictive capacity of the reduction in mREE at 12 months from baseline for: **A** EWL<50% at nadir and **B** weight regain after 5 years follow-up

We found no significant pre-BS predictors of reduction in m REE at 1m and 12m follow-up, among age, gender, BS technique, and obesity-related comorbidities. These parameters neither were predictors of significant weight regain at 5 years follow-up. Interestingly, the reduction of mREE at 12 months (calculated as mREE-baseline − mREE-12m) was a significant predictor of the following: (A) poor nadir weight loss after BS (%EWL<50%) and (B) weight regain at 5 years follow-up (AUCROC of 0.841 (95%CI [0.655–0.909], *p*=0.032) and AUCROC of 0.855 (95% CI [0.639–0.901]), *p*= 0.027, respectively) (Figure 3).

## Discussion

In the present study, we showed for the first time that an early and significant reduction in the REE (evaluated by means of IC-gold standard method) occurs in patients with SO that undergo bariatric surgery, up to levels comparable to those of the normoweighted Spanish population [27], despite the fact that 1 month after BS, their BMI is still in morbid obesity range. Furthermore, in our study, we showed that the reduction in REE at 12 months after the BS was a good predictor of a “good” or “poor” response to BS (“good” defined as %EWL nadir>50%) with a AUCROC of 0.841 (95%CI [0.655–0.909], *p*=0.032) as well as for weight regain after 5 years of follow-up with an AUCROC of 0.855 (95% CI [0.639–0.901], *p*= 0.027). In other words, the greater the reduction in REE 1 year after BS, the less %EWL at nadir and the greater the weight regain after 5 years*.*

As explained in the “Introduction,” at present there is no reliable data on basal REE in patients with SO. We found in the literature only one study [28] that used IC, to compare our results at baseline and showed similar results (mean REE 2262 ± 122 kcal/day in patients with BMI 56kg/m^2^). Most of the studies published so far in patients with obesity and most of the few studies that were reported on SO used an estimated value of REE by means of equations [20]. These equations were validated and calculated based on standard adults with normal weight [9]. They might not be adequate for patients with obesity, and in particular with SO, and at present this represents an important gap in the personalized management of these patients. A recent external validation of REE predictive equations reported that the accuracy of the formulas decreases going from normal weight to class 3 obesity [29]. Having a real characterization of the REE in this population is necessary in order to personalize the diet (in particular calorie intake) and to assure a safe and effective weight loss and, more importantly, weight maintenance after successful weight loss, although compliance was shown to be limited in the case of long-lasting calorie-restriction intake [30].

In order to shed light on this gap, in our study, we compared the values of the REE estimated by the standard recommended equations and the gold standard method, the indirect calorimetry. We found that at baseline the MSJ equation significantly underestimated the REE when compared to the gold standard (IC) (1994.44 ± 463.41 vs 2320.4 ± 750.8, *p*=0.031). In exchange, after the BS, we found that the MSJ overestimated REE in males (2194.80±177.32 kcal/day vs 1555.73±40.40kcal/day, *p*<0.001), while in females showed no significand difference when compared to the REE measured by IC. Additionally, although we found a significant difference between gender at mREE-baseline, these differences were no longer significant after BS, while MSJ showed differences between gender in all three time points (baseline, 1m, and 12m). This is an interesting finding and highlights the limitations of these equations that do not take into the account all the particularities of the patients with morbid obesity and in particular with SO. The MSJ estimates the REE by including the gender into the formula, but this formula was calculated using data from standard adults with normal weight and probably normal body composition [25]. A possible explanation of this overestimation of eREE in males after the BS is that the formula of the equation does not include data on the changes that occur in body composition, in particular muscle mass loss after the BS [31]. A significant reduction in muscle mass after the BS might explain the differences between the overestimated REE by equations and the real REE measured by IC.

Additionally, in our study, we found a significant reduction in mREE very early after the BS and 1 month and remained unchanged after 12 months, at similar levels with normoweighted Spanish population. Mean mREE-1m: 1537.67 ± 83.67 kcal/day, similar to the mREE of 1589±312 kcal/day found by De la Cruz et al. in healthy individuals with normal weight in Spain [27]. Furthermore, the change in mREE from baseline to 12 months was a significant predictor of successful weight loss after BS and weight regain after 5 years follow-up (AUCROC of 0.841 (95%CI [0.655–0.909], *p*=0.032) and AUCROC of 0.855 (95% CI [0.639–0.901]), respectively), *p*= 0.027, respectively). No other factor included in the analysis showed a significant predictive value of evolution after BS (age, gender, BS technique, obesity-related comorbidities). Moreover, we found no differences in REE between the two types of surgery performed at any time points. However, it should be noted that the study design was not powered to find these differences.

Additionally, an indirect correlation was observed between mREE-1m and mREE-12m and between RQ-1m and RQ-12m, but not with mREE and RQ at baseline. This finding suggests a metabolic adaptation after BS or a state of altered energy balance in the time points after surgery that can offer a partial explanation of the role of these changes in weight loss and weight regain after the BS. Metabolic adaptation (MA) is defined as the residual eREE after adjusting for changes in body composition and age [13]. Although a negative energy balance, whether due to a decrease in caloric intake or an increase in energy consumption, would result in weight loss, it has been proposed that the weight loss activates compensatory mechanisms that condition the decrease observed in REE after surgery [32]. Previous data in the literature suggested that a greater than predicted drop in mREE after an intervention induces a metabolic adaption, independently of the fat-free mass [33]. These data, and data from our study, indicate that maybe significant changes in muscle mass that occur after BS can play a crucial role in the evolution of REE and evolution after the BS in terms of weight loss and maintenance.

Our study has several limitations: (A) REE alone was measured, rather than total energy expenditure, which includes DIT and AEE. Although REE accounts for around 70% of total energy expenditure under normal circumstances, the changes in REE associated with weight loss parallel those in total energy expenditure [34]. (B) Lack of body composition evaluation and (C) evaluation of dietary intake.

### Concluding Remarks

The validated equations used widely in the clinical practice are not reliable for the REE estimation in patients with SO. We showed for the first time that in patients with SO, a significant reduction of the REE occurs at 1 month after the BS, remains unchanged at 12 months, and is the major conditioning of successful weight loss and maintenance after the BS. Further studies are needed in order to shed light on these data, and to explore the underlying mechanisms.
